# A clinically applicable tool for rapidly estimating muscle volume using ultrasound images

**DOI:** 10.1007/s00421-019-04242-2

**Published:** 2019-10-25

**Authors:** Daniel T. Rothwell, Daniel T. P. Fong, Sarah A. Stapley, David J. Williams

**Affiliations:** 1 National Centre for Sport and Exercise Medicine, School of Sport, Exercise and Health Sciences, Loughborough University, Loughborough, UK; 2 Academic Department of Military Surgery and Trauma, Royal Centre for Defence Medicine, Birmingham, UK; 3 Wolfson School of Mechanical, Electrical and Manufacturing Engineering, Loughborough University, Loughborough, UK

**Keywords:** Ultrasound, Muscle group, Muscle size, Clinical application, Musculoskeletal imaging

## Abstract

**Purpose:**

This study aimed to identify a safe, rapid, and accessible method of estimating muscle volume of key lower limb muscle groups to reduce the time-demand of acquiring this measurement and potentially facilitate its application as a clinical monitoring tool.

**Methods:**

Unilateral MRI images were acquired from the 12th thoracic vertebrae to the base of the foot in 18 recreationally active males. Panoramic B-mode ultrasound images were acquired from the same leg at the mid-hip, 25%, 50%, and 75% of thigh length, and 25% of shank length. Body mass, height, limb lengths, and circumferences at the sites corresponding to the ultrasound images were acquired. A single investigator manually analysed all images. Regression analyses were conducted to identify models for estimating volume of the hip extensor, knee extensor and flexor, and ankle plantarflexor muscle groups.

**Results:**

Models were developed for estimating hip extensor (SEE = 8.92%, *R*^2^ = 0.690), knee extensor (SEE = 5.24%, *R*^2^ = 0.707) and flexor (SEE = 7.89%, *R*^2^ = 0.357), and ankle plantarflexor (SEE = 10.78%, *R*^2^ = 0.387) muscle group volumes. The hip and knee extensor models showed good potential for generalisation. Systematic error was observed for the knee flexor and ankle plantarflexor models.

**Conclusions:**

Hip extensor, knee extensor and flexor, and ankle plantarflexor muscle group volumes can be estimated using B-mode ultrasound images and anthropometric measurements. The error shown for each of the models was sufficient to identify previously reported differences in muscle volume due to training or injury, supporting their clinical application.

## Introduction

Evidence is available supporting a positive relationship between muscle size and strength (Evangelidis et al. 2016), although strength improvements have been observed independently of changes in muscle size (Jessee et al. 2018). Nonetheless, muscle size is a useful variable providing insight into a patient or athlete’s physical condition, and can identify areas of focus for training and rehabilitation, as well as being valuable for quantifiably monitoring progress. Therefore, providing clinicians with the ability to rapidly obtain a measurement of muscle size can facilitate the application of muscle size as a monitoring tool during training and rehabilitation.

Muscle size is most commonly quantified as muscle volume (MV). This is a more consistent measurement and is less dependent on the image acquisition process in comparison to cross-sectional area (CSA) and muscle thickness (MT) measurements, as the volume of a muscle should remain constant when injury associated atrophy or training associated hypertrophy is not present. Atrophy of muscles and groups in injured populations has previously been reported in the hip extensors (Jaegers et al. 1995; Grimaldi et al. 2009a, b), knee flexors and extensors (Mizner et al. 2005; Almurdhi et al. 2016), and ankle plantarflexors (Almurdhi et al. 2016; Feger et al. 2016; Handsfield et al. 2016). These muscle groups are important for supporting the body during locomotion (hip and knee extensors, and ankle plantarflexors) and providing lower limb stability (knee flexors). Previous research has also found that MV of these groups is larger in elite athletes compared to controls (Semciw et al. 2016; Handsfield et al. 2017). Differences in lower limb MV have been found between athletes from different sports (Bex et al. 2017) and small differences have been observed between legs in athletes from sports with a leg dominance (Tate et al. 2006). Muscle volume has been found to increase with specific training in the knee extensors (Balshaw et al. 2017), giving this measurement a role in the monitoring of training and rehabilitation programmes.

Muscle volume is typically calculated in vivo by manually analysing images acquired from Magnetic Resonance Imaging (MRI). This analysis is both cost- and time-intensive, limiting its application in clinical and research environments. Indeed, the time-demand for manually analysing the entire lower limb has been reported to be in excess of 24 h (Handsfield et al. 2014; Rothwell et al. 2019). The partial volume of a 100 mm section of the knee extensors has been associated with knee extensor strength in patients with a history of anterior cruciate ligament reconstruction (Kuenze et al. 2016) and using partial volumes to represent MV may be a viable option. Evidence is also available to suggest that analysing a reduced number of axial images can provide an estimate of MV with good agreement and minimal error compared to a rigorous criterion (Tracy et al. 2003; Morse et al. 2007; Cotofana et al. 2010; Hogrel et al. 2015; Vanmechelen et al. 2018). However, the operating costs associated with MRI limit its use on a regular basis, and it is not possible to acquire images from some individuals, such as those with known or suspected metal objects in their body, due to the heating effect of being in the magnetic field (e.g., individuals with shrapnel, orthopaedic implants such as pins or plates, or pacemakers). As well as reducing the time-demand and cost of obtaining an estimate of MV, the safety of application in a clinical environment is of paramount importance.

Brightness-mode (B-mode) ultrasound is a safe, non-ionising, and accessible tool for use in measuring lower limb MV. It has been used to acquire multiple axial images, similar to those obtained using MRI, allowing CSA to be measured and used to calculate MV, although the time requirement of more than 40 min per participant does not support clinical application (Esformes et al. 2002; Scott et al. 2012). Acquiring a single ultrasound image and taking a single measurement, such as MT (Miyatani et al. 2003) or CSA (Park et al. 2014), and multiplying this by limb length have previously shown good application for the knee extensors and gastrocnemii, respectively, although the final models were not cross-validated and generalisation is unknown. Models developed using regression analyses tend to be population specific and those developed in young (Miyatani et al. 2004) perform poorly in older populations (Nakatani et al. 2016), likely due to increased intramuscular adipose tissue with age (Yoshiko et al. 2017), resulting in different muscle shapes and altering the relationship between transverse measurements such as MT and CSA, and MV. To date, research using ultrasound to estimate MV has been limited to the quadriceps and plantarflexors, likely because, in addition to the functional importance these muscles, they are straightforward to identify from ultrasound images in comparison to the hip extensors and knee flexors. Indeed, all previous studies using ultrasound imaging to measure hamstring muscle size have used MT rather than CSA (Weiss 1984; Thoirs and English 2009; English et al. 2012) and no studies have used ultrasound measurements to estimate the volume of the knee flexor and hip extensor muscle groups. A significant omission from previously developed models is body mass which has shown a strong correlation with MV (Handsfield et al. 2014), making it a variable worthy of consideration, particularly due to the role of the hip and knee extensors and ankle plantarflexors in supporting body weight during locomotion and weight-bearing activities.

Providing clinicians and researchers with a safe and valid tool for rapidly estimating the volume of key lower limb muscle groups such as the hip extensors, knee flexors and extensors, and ankle plantarflexors, could lead to the inclusion of MV in the regular assessment and longitudinal monitoring of patient and athlete progress. This can assist in identifying the onset of pathology or risk of injury, leading to early intervention and improved management of musculoskeletal injuries. This study contributed to the development of a straightforward clinical tool for rapidly measuring the volume of functionally important lower limb muscle groups, namely the hip extensors, knee flexors and extensors, and ankle plantarflexors. This was achieved by conducting regression analyses with B-mode ultrasound images and anthropometric measurements and conducting cross-validation to identify the potential of the models for generalisation. It was hypothesised that the most accurate and generalisable models would be developed for the knee extensors and ankle plantarflexors as the identification of muscle boundaries in these groups is more straightforward than for the knee flexors and hip extensors. Similarly, measuring the volume of the hip extensors and knee flexors was hypothesised to be challenging due to difficulty in identifying muscle boundaries. Body mass was hypothesised to be an important variable for the models developed for the hip and knee extensor and ankle plantarflexor groups as these muscles provide body weight support during locomotion and weight-bearing activities.

## Methods

### Participants

After gaining university ethical approval, healthy Caucasian males were recruited by word of mouth and poster advertisements. Eighteen participants gave informed consent to participate in the study (age 29.0 ± 5.6 years, height 1.81 ± 0.07 m, and body mass 79.8 ± 10.9 kg). The mean height and body mass of the model-development and cross-validation groups for each muscle group are reported in Tables 2, 3, 4, and 5. All participants were healthy, free from injury for a minimum of 3 months prior to the study and were participating in recreational exercise for a minimum of 30 min per week three times per week. All assessments were conducted on the preferred limb which was defined as that used to balance on one leg or stop oneself from falling when pushed from behind. Fourteen participants preferred their right leg and four preferred their left.

### Anthropometric measurements

For all participants, anthropometric measurements were taken before MRI and ultrasound imaging. After measuring height (metres, m) and body mass (kilograms, kg), participants lay supine on a treatment plinth and length measurements (m) of the thigh (from the greater trochanter to the lateral epicondyle of the femur) and shank (from the lateral epicondyle of the femur to the lateral malleolus) were acquired using anthropometric callipers (seca 207, Seca GmbH, Germany). The distance from the greater trochanter to 25%, 50%, and 75% of thigh length, and from the lateral epicondyle of the femur to 25% of shank length was marked on the skin as a horizontal line with permanent marker pen on the anterior, posterior, medial, and lateral aspects of the limb to assist both anthropometric and ultrasound measurement acquisition. Limb circumferences (cm) were measured at each of these four sites with a tape measure and an additional circumference of the hips was measured at the level of the greater trochanter and multiplied by 0.5 to represent half of the hip circumference.

### MRI data acquisition

Unilateral axial spin-echo T1-weighted MRI images were acquired from the twelfth thoracic vertebrae (or the level corresponding to the origin of psoas major, verified by an experienced radiographer) to the base of the foot of the preferred leg using 3-Tesla MRI (Discovery MR750w, GE Healthcare, General Electric, Boston, MA, USA). Images were acquired in four or five scanning blocks of 34–86 slices depending on participant height. Block overlap was identified using fish oil capsule references which were visible on the images used in the analysis. A single fish oil capsule reference was also placed at the 25%, 50%, and 75% of thigh length and 25% of shank length sites to enable these to be identified in the analysis. Slice thickness was 5 mm for all participants; inter-slice distance was 0 mm for all participants apart from one where the inter-slice distance was 5 mm. The in-plane resolution for all images was 0.47 mm × 0.47 mm. Echo time (TE = 7.546–16.940 ms) and repetition time (TR = 533–845 ms) varied between scanning blocks for the first four participants and remained constant between scanning blocks for the remaining fourteen participants. The first, most proximal, image block was an exception to this and the optimal TE and TR were chosen by the radiographer to minimise movement artefact in the abdominal region due to breathing. Field of view (144 mm × 144 mm to 450 mm × 450 mm) and flip angle (90º–111º) were varied by the radiographer to obtain the best quality image for each participant in each scanning block. Total scanning time was approximately 50 min per participant including breaks between scanning blocks.

### Ultrasound imaging data acquisition

Panoramic B-mode ultrasound images were acquired by a single investigator using a Logiq E9 ultrasound scanner (GE Healthcare, General Electric, Boston, MA, USA) with a 44 mm, 2–8 MHz 9L linear-array transducer, coated in water-soluble transmission gel which enabled acoustic contact without depression of the skin and superficial adipose tissue. Participants lay on a treatment plinth at rest, while images were acquired at the mid-hip, and at 25%, 50%, and 75% of thigh length, and 25% of shank length.

The mid-hip site was identified in side-lying, on the contralateral side to that being imaged, with the hip and knee of the bottom leg flexed, so that a standardised, comfortable, and relaxed position could be maintained. The vertical distance between the iliac crest and the greater trochanter was measured with a measuring tape and the mid-way point was marked with pen. A perpendicular line was drawn, along which a panoramic image was acquired, starting at the anterior superior iliac spine and moving posteriorly towards the sacrum.

Images of the anterior aspect of the thigh were acquired with participants sat in a recumbent position on the treatment plinth with a foam roller positioned under their knees to ensure consistency between measurements, in ~ 10º knee flexion. The transducer was positioned on the lateral side of the leg, perpendicular to the skin in the transverse plane, and moved medially as far as possible without losing contact with the skin or changing the angle of the probe against the skin. This process was repeated for all the anterior sites and in participants with large thighs two images were necessary to ensure that all muscles were acquired. After acquiring images from all the anterior sites, participants lay prone with their toes hanging over the edge of the plinth and images were acquired from the posterior aspect of the thigh and shank sites. Images were reviewed on the ultrasound machine visual display after collection and repeated when required to ensure that muscle CSA could be clearly identified in a single image.

### Data processing—image analysis

All MRI and ultrasound images were analysed in OsiriX Lite (v.8.0.1, Pixmeo, Geneva, Switzerland) open source software by a single experienced investigator. The brightness and zoom tools were used to improve tissue contrast and enable muscle boundaries to be better identified. The boundaries of the individual hip extensor (gluteus maximus and medius, biceps femoris long head, semimembranosus, semitendinosus, and adductor magnus) knee extensor (rectus femoris, vastus intermedialis, lateralis, and medialis) and flexor (semimembranosus, semitendinosus, biceps femoris long and short heads, medial and lateral gastrocnemius, popliteus, sartorius, and gracilis), and ankle plantarflexor muscles (medial and lateral gastrocnemius, soleus, peroneals, tibialis posterior, flexor digitorum longus, and flexor hallucis longus) were manually outlined at intervals of 15 mm on the MRI images using a graphics tablet (XP-Pen, XPPEN Technology CO, Fullerton, CA, USA). For the data set with 5 mm spacing, larger muscles such as the quadriceps, hamstrings, and gluteus maximus were analysed with an inter-slice distance of 20 mm and smaller muscles were analysed with an inter-slice distance of 10 mm, as a preliminary analysis in three participants suggested this maintained measurement accuracy and reduced the analysis time compared to using an inter-slice distance of 5 mm. The resulting CSA measurements (in centimetres squared, cm^2^) were used in the calculation of MV.

For ultrasound images, CSA of the individual quadriceps (rectus femoris, vastus medialis, lateralis, and intermedialis), hamstrings (biceps femoris long and short head, semitendinosus, and semimembranosus), and gastrocnemii (lateral and medial gastrocnemius, and soleus) were outlined on the respective images. Ultrasound images acquired at the hip were not used to measure CSA. Superficial muscles were chosen as they provide the best potential for generalisation of the final model, as they are more likely to be consistently identifiable in the wider population. The sum of the quadriceps and hamstrings CSA at 50% and 75% of thigh length was calculated for inclusion in the regression analyses.

**Fig. 1:**
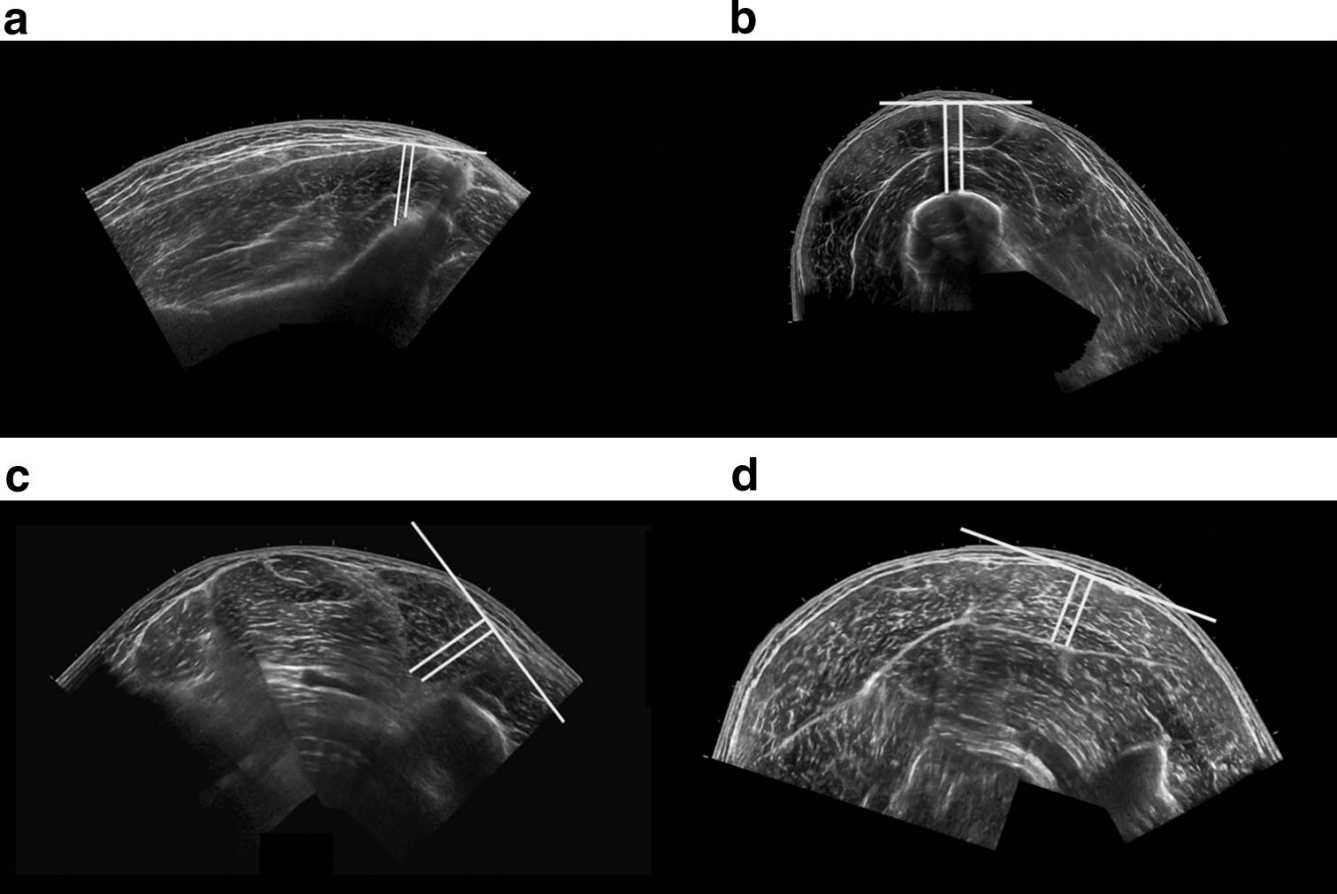
Ultrasound muscle thickness measurements of **a** the gluteals, **b** the anterior thigh at 50% of thigh length, **c** biceps femoris short head on the posterior thigh at 75% of thigh length, and **d** lateral gastrocnemius on the posterior shank at 25% of shank length. The white lines indicate the measurements used to represent muscle thickness

Muscle thickness (MT) measurements were taken by drawing two parallel lines perpendicular to a straight line on the superficial aponeurosis of each muscle, extending to the deep aponeurosis (Fig. 1). The mean value of the length of the two perpendicular lines was taken as MT (in centimetres, cm). As considerable training and experience are necessary to acquire valid ultrasound images of the hip extensors, limiting the potential for practical application, the only ultrasound measurement acquired at the hip was a single MT measurement (including gluteus medius and minimus) to facilitate the potential for clinical application of the findings (Fig. 1a). Single measurements of MT at 25% and 50% of the anterior (including rectus femoris and vastus intermedialis) and lateral thigh (including vastus lateralis and vastus intermedialis), and at 25% of the medial (including medial gastrocnemius and soleus) and lateral (including lateral gastrocnemius and soleus) triceps surae were also acquired for inclusion in the regression analyses to potentially assist in reducing image acquisition time. A single MT measurement to represent the hamstrings was not acquired as this is a superficial muscle group and the underlying adductor magnus does not contribute to knee flexion. To evaluate the application of an MT measurement representing all the individual quadriceps and hamstrings muscles in the respective knee extensor and flexor groups, the sum of the individual MT measurements at 50% and 75% of thigh length was also calculated. The reliability of ultrasound and MRI CSA and MT measurements was evaluated between sessions in two randomly selected participants with a minimum of 7 days between sessions.

The criterion MV was calculated using MRI CSA of the individual hip extensor, knee extensor and flexor, and ankle plantarflexor muscles. The volume of individual muscles (in centimetres cubed, cm^3^) was calculated using the formula:

$$\mathop \sum \limits_{i = 1 \ldots n - 1}^{n} \left( {\frac{{{\text{CSA}}_{i} \; + \; {\text{CSA}}_{i + 1} }}{2}} \right) \; \times \; h,$$

where CSA_*i*_ is CSA at slice *i*, CSA_*i*+1_ is CSA at slice *i *+1, *h* = distance between slices, and *n* = total number of slices in the muscle. Individual MV was summed to calculate group MV (i.e., hip extensors, knee extensors and flexors, and ankle plantarflexors).

### Statistical analysis

All computational analyses were completed in Microsoft Excel 2010 (Microsoft Inc., Redmond, WA, USA). Measurement reliability of ultrasound CSA and MT measurements was calculated as the typical error of measurement (TEM) between sessions:

$${\text{TEM}}\; = \;\frac{{{\text{SD}}_{\text{differences}} }}{\sqrt 2 },$$

where SD_differences_ is the muscle-specific standard deviation of the differences between sessions. TEM are presented as absolute values and as a percentage of the mean value of the first measurement.

All statistical analyses were conducted in IBM SPSS Statistics 23 (IBM Corporation, Armonk, NY, USA) and the alpha level for statistical significance was set to *p* < 0.05. Kolmogorov–Smirnov tests of normality showed a normal distribution for MV of the ankle plantarflexors. The knee extensors (*p* = 0.009) and knee flexors (*p* = 0.041) were not normally distributed due to two outlying participants who were not included in the regression analyses for these muscle groups, to ensure integrity in the final models developed. The hip extensors (*p* = 0.011) were not normally distributed due to one outlying participant who was not included in the regression analyses for this muscle group. All anthropometric variables were normally distributed. All ultrasound CSA measurements were normally distributed, and all MT measurements were normally distributed with the exception of semitendinosus at 75% of thigh length (*p* = 0.040) and as this was a small measurement, and semitendinosus was also represented at 25% and 50% of thigh length, it was excluded from the regression analysis.

Regression analyses were conducted for estimating the volume of each muscle group on a level-wise (using all measurements at 25%, 50%, or 75% of limb length) and muscle-wise (using all measurements for individual muscles) basis using all ultrasound and anthropometric measurements. Regression analyses were conducted separately for CSA and MT measurements to prevent collinearity of independent variables. To identify the most appropriate independent variables to include in the regression analysis, semi-partial correlations between all potential independent variables and the dependent variable (the respective MV) were conducted using the data of all participants, excluding outliers. The independent variable with the strongest semi-partial correlation with MV was chosen for the regression analysis to allow an approximate ratio of one independent variable for every 15 cases, as recommended by Field (2013). If two independent variables had a similar strength semi-partial correlation, the Pearson’s product moment correlation between these independent variables was calculated to evaluate collinearity and if this was present, the independent variable with the weakest semi-partial correlation was excluded from further analyses. If collinearity was not present, both independent variables were included in the analysis.

For the regression analysis, the sample was randomly divided into sub-samples of 20% (*n* = 3 for hip extensors and knee extensors and flexors; *n* = 4 for ankle plantarflexors) and 80% (*n* = 14 for hip extensors and ankle plantarflexors; *n* = 13 for knee extensors and flexors) as recommended by Field (2013). A *k*-fold leave-one-out cross-validation was conducted using forced entry regression to identify appropriate independent variables for the final models. The suitability of the models developed was evaluated based on the following diagnostic criteria: (1) to evaluate the generalisation of the model; the 95% confidence intervals for the beta coefficients did not cross zero, no more than one participant had a standardised residual greater than ± 2.00, and the *R*^2^ and adjusted *R*^2^ values were similar; (2) to ensure that all independent variables significantly contributed to the model; the *p* values of all beta coefficients were less than 0.05; (3) to ensure the absence of collinearity, the independent variables were not strongly correlated (i.e., > 0.80); (4) to evaluate the assumption of additivity; the semi-partial correlations between all independent variables and the dependent variable were similar; (5) to evaluate the assumption of homoscedasticity; there was no positive or negative trend in the plot of standardised residuals against standardised predicted values; (6) to test the assumptions of independent and normally distributed errors, the Durbin–Watson statistic was above the upper limits identified by Durbin and Watson (1951) and the plot of residuals was normally distributed; and (7) the ease and speed of acquisition and measurement reliability were considered when selecting the final model in the context of clinical application.

Independent variables were identified as appropriate when they were present in all *k*-fold analyses for each respective muscle group. The identified independent variables were included in a forced entry regression analysis to identify the final model using the data of all participants in the 80% sub-sample. The equation was cross-validated in the 20% sub-sample and the final model was evaluated using the same diagnostic criteria described above. When the final model included more than one variable, its performance in simple and multiple regression analyses was evaluated, and if both showed good potential for generalisation, the bivariate correlation between the residuals of each model was calculated to identify the statistical difference between models and assist in selecting the final model. When a strong correlation was observed between residuals of multiple and simple regression models, the final model selected was that with the highest *R*^2^ value. For the final models, the systematic difference was examined by plotting the residuals (MRI-derived MV minus model-derived MV) against the mean value of the estimated and actual MV for all participants (Bland and Altman 1986).

## Results

### Regression analysis

One model was identified for the hip extensors and ankle plantarflexors and multiple models were identified for the knee flexors and extensors. Table 1 summarises how the final model was systematically selected for each muscle group.

**Table 1:**
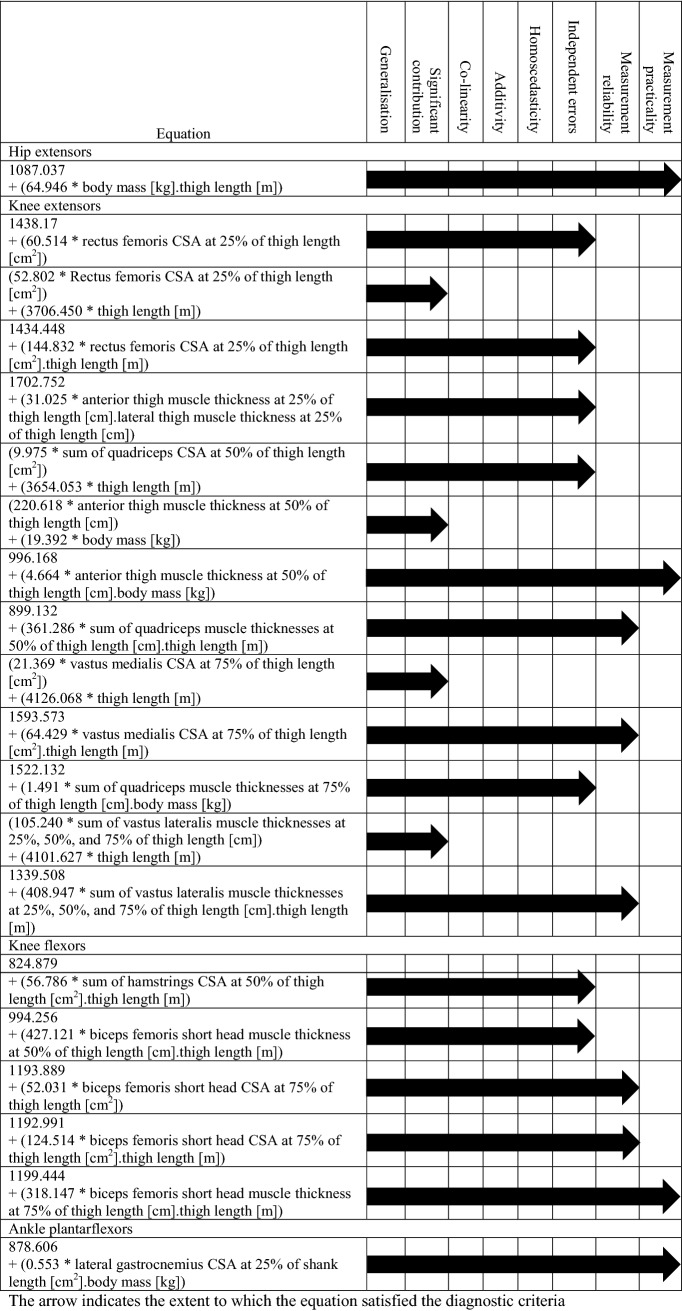
Summary of the selection of the final regression model for the hip extensors, knee extensors and flexors, and ankle plantarflexors

### Hip extensors

**Fig. 2:**
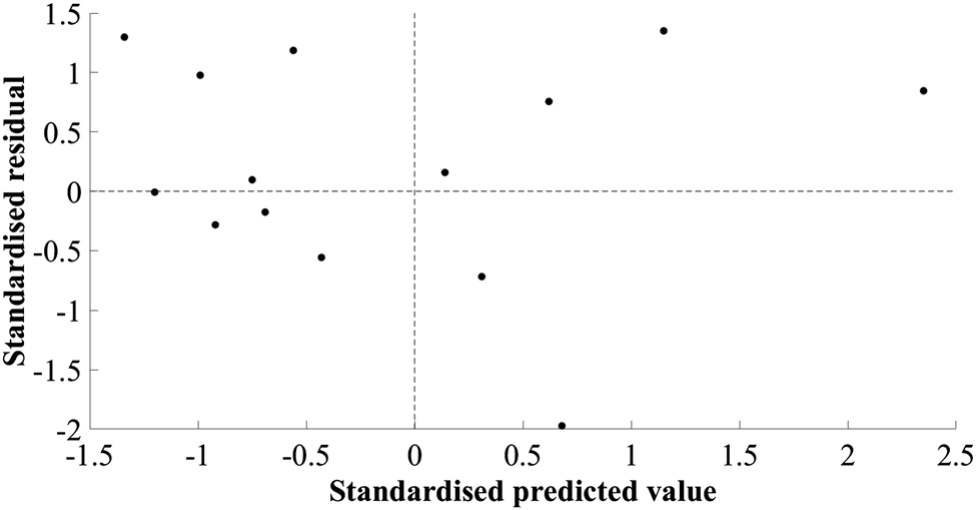
Scatter plot of standardised residuals versus standardised predicted values for the final hip extensor model; 1087.037 + (64.946 × body mass.thigh length)

**Fig. 3:**
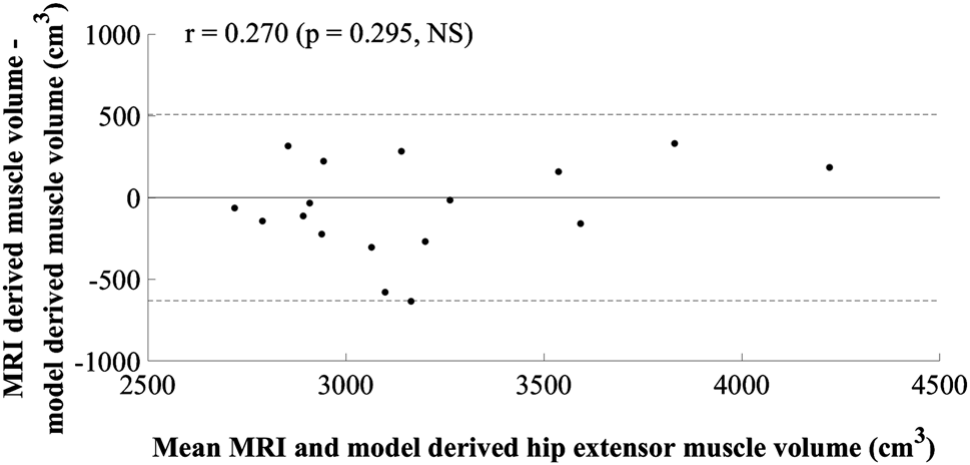
Mean value of hip extensor muscle volume calculated using MRI and the newly developed model plotted against the difference between methods

The model developed for estimating MV of the hip extensors did not include a measurement of muscle size. The final simple regression model included the product of body mass and thigh length, in kilograms and metres, respectively (*R*^2^ = 0.690, SEE = 283.01 cm^3^, 8.92%). A large residual was observed in cross-validation (mean cross-validation residual = − 347.32 cm^3^, − 11.28%, Table 2). The adjusted *R*^2^ value, 0.665, was similar to the *R*^2^ value, 0.690, indicating good application of the model to the population. The plots of standardised predicted versus standardised residual values showed that assumptions of linearity and additivity, homoscedasticity, and normally distributed residuals had been met (Fig. 2). The Durbin–Watson statistic was 1.67 in the model-development group, which was above the upper limit of 1.40 reported by Durbin and Watson (1951), supporting the absence of autocorrelation of residuals. A multiple regression model resulted in 95% confidence intervals crossing zero and was not suitable for generalisation. The mid-hip MT measurement had a weak semi-partial correlation and attempts to include this in the model resulted in poor generalisation. The plot of residuals versus the mean MV showed the absence of systematic differences between estimated and actual MV (*r* = 0.270, *p* = 0.295, Fig. 3).

**Table 2:** Hip extensor muscle group regression equation, model parameters, diagnostic statistics, and group characteristics (mean ± standard deviation)

Equation: ${\text{Hip extensor muscle volume}}\; = \; 10 8 7.0 3 7\; + \;\left( { 6 4. 9 4 6\; \times \;{\text{body mass}}.{\text{thigh length}}} \right)$			
	*β*	Standard error (lower limit–upper limit)	*p* value
Constant	1087.037	410.022 (193.676–1980.398)	0.021
Body mass.thigh length	64.946	12.554 (37.594–92.298)	< 0.001
*R*^2^	0.690		
Adjusted *R*^2^	0.665		
Standard error of the estimate (cm^3^)	283.01 (8.92%)		
Mean cross-validation residual (cm^3^)	− 347.32 (11.28%)		
Durbin–Watson statistic	1.67		

	Model-development group	Cross-validation group (range)
Height (m)	1.78 ± 0.05	1.88 (1.80–1.93)
Body mass (kg)	77.8 ± 9.7	80.3 (75.4–84.7)
Thigh length (m)	0.41 ± 0.03	0.45 (0.41–0.47)
Hip extensor muscle volume (cm^3^)	3171.9 ± 488.7	3078.8 (2810.1–3513.3)

Model-development group *n* = 14, cross-validation group *n* = 3

### Knee extensors

**Fig. 4:**
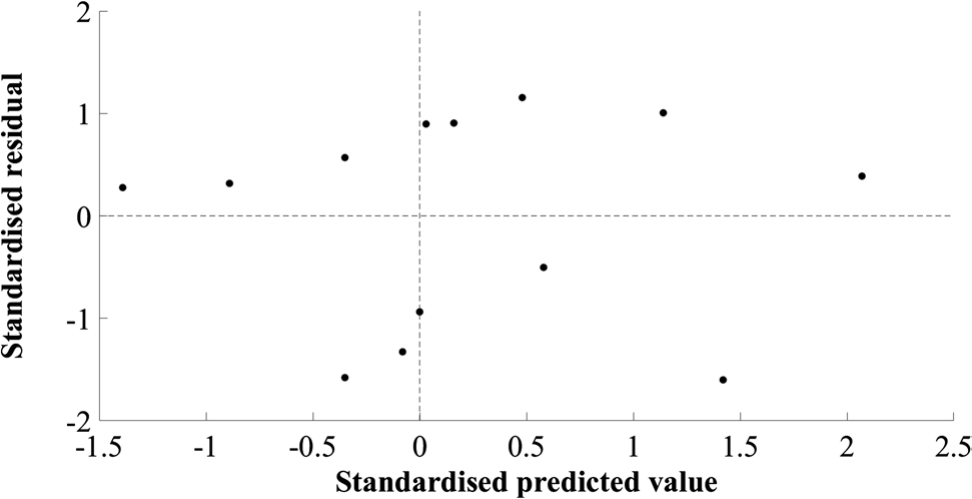
Scatter plot of standardised residuals versus standardised predicted values for the final knee extensor model; 996.168 + (4.664 × anterior thigh muscle thickness at 50% of thigh length.body mass)

**Fig. 5:**
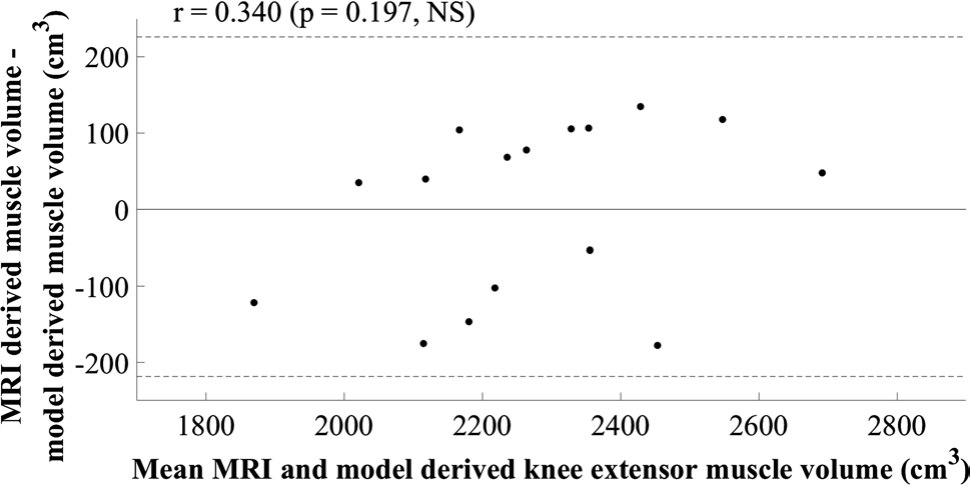
Mean value of knee extensor muscle volume calculated using MRI and the newly developed model plotted against the difference between methods

The final equation selected for the knee extensors’ volume estimation model was a simple regression equation including the product of anterior thigh MT at 50% of thigh length and body mass, in centimetres and kilograms, respectively (Table 3). A multiple regression model resulted in multicollinearity between MT and body mass, as was also the case for the other multiple regression models evaluated for the knee extensors (Table 1). The final model provides good potential for clinical application as the measurement of anterior thigh MT is not limited by the size of the ultrasound probe or the anatomical knowledge of the clinician. Figure 4 shows that the assumptions of linearity and additivity, homoscedasticity, and normally distributed residuals were met. The SEE for the model was 121.10 cm^3^, 5.24%, *R*^2^ was 0.707, and adjusted *R*^2^ was 0.681. The cross-validation residual was 20.11 cm^3^, 0.95%, and the Durbin–Watson statistic, 2.32, was above the upper limit described by Durbin and Watson (1951), supporting the independence of errors. The plot of residuals versus the mean value showed a slight positive trend, although the correlation observed was not significant (*r* = 0.340, *p* = 0.197, Fig. 5).

**Table 3:** Knee extensor muscle group regression equation, model parameters, diagnostic statistics, group characteristics (mean ± standard deviation), and between-session reliability of ultrasound measurements

Equation: ${\text{Knee extensor muscle volume}}\; = \;996.168\; + \;\left( {4.664\; \times \;{\text{anterior thigh muscle thickness at }}50\% {\text{ of thigh length}} . {\text{body mass}}} \right)$			
	*β*	Standard error (lower limit – upper limit)	*p* value
Constant	996.168	257.349 (429.747–1562.588)	0.003
Anterior thigh muscle thickness at 50% of thigh length.body mass	4.664	0.905 (2.673–6.655)	< 0.001
*R*^2^	0.707		
Adjusted *R*^2^	0.681		
Standard error of the estimate (cm^3^)	121.10 (5.24%)		
Mean cross-validation residual (cm^3^)	20.11 (0.95%)		
Durbin–Watson statistic	2.32		

	Model-development group	Cross-validation group (range)	Between-session difference (TEM)
Height (m)	1.81 ± 0.07	177.1 (171.5–181.2)	
Body mass (kg)	78.2 ± 8.2	72.7 (65.7–80.5)	
Thigh length (m)	0.42 ± 0.04	0.41 (0.39–0.43)	
Anterior thigh muscle thickness at 50% of thigh length (cm)	3.6 ± 0.4	3.7 (3.0–4.4)	0.1 (2.0%)
Knee extensor muscle volume (cm^3^)	2311.6 ± 214.3	2110.32 (1809.1–2302.9)	

Model-development group *n* = 13, cross-validation group *n* = 3

### Knee flexors

**Fig. 6:**
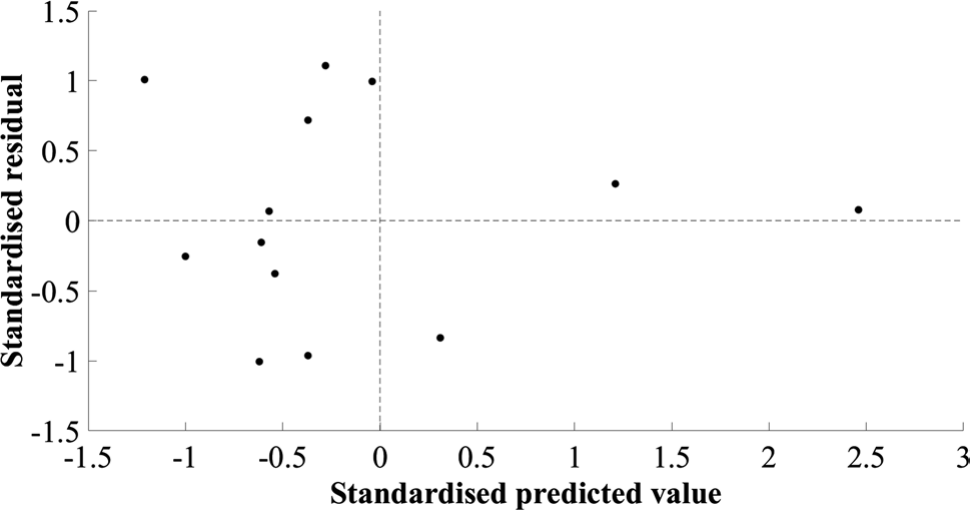
Scatter plot of standardised residuals versus standardised predicted values for the final knee flexor model; 1199.44 + (318.147 × biceps femoris short-head muscle thickness and 75% of thigh length.thigh length)

**Fig. 7:**
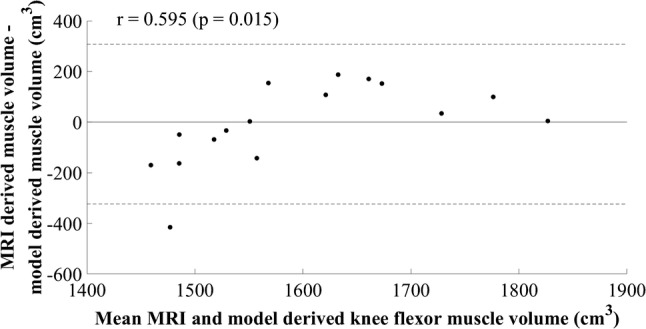
Mean value of knee flexor muscle volume calculated using MRI and the newly developed model plotted against the difference between methods

The final model identified for the knee flexors was a simple regression model (Table 1), using a constant and the product of biceps femoris short-head MT at 75% of thigh length and thigh length, in centimetres and metres, respectively (Table 4). The SEE was 125.37 cm^3^, 7.89%, *R*^2^ was 0.357, and adjusted *R*^2^ was 0.298, suggesting good generalisation of the model, although the *R*^2^ values were low. The cross-validation residual was − 42.72 cm^3^, − 2.66%. Implementing the final model using multiple regression resulted in 95% confidence intervals for the constant and the beta coefficients crossing zero, limiting generalisation. The final model selected was the simple regression model. The assumptions of linearity and additivity, homoscedasticity, and normal distribution of errors were met (Fig. 6), and the Durbin–Watson statistic confirmed the absence of autocorrelation between residuals. The plot of residuals versus the mean value showed a statistically significant positive trend (*r* = 0.595, *p* = 0.015, Fig. 7).

**Table 4:** Knee flexor muscle group regression equation, model parameters, diagnostic statistics, participant characteristics (mean ± standard deviation), and between-session reliability of ultrasound measurements

Equation: ${\text{Knee flexor muscle volume}}\; = \; 1 1 9 9. 4 4 4\; + \;\left( { 3 1 8. 1 4 7\; \times \;{\text{biceps femoris short head muscle thickness at 75}}\% {\text{ of thigh length}}.{\text{thigh length}}} \right)$			
	*β*	Standard error (lower limit – upper limit)	*p* value
Constant	1199.444	161.753 (843.428–1555.461)	< 0.001
Biceps femoris short-head muscle thickness at 75% of thigh length.thigh length	318.147	128.860 (34.528–601.766)	0.031
*R*^2^	0.357		
Adjusted *R*^2^	0.298		
Standard error of the estimate (cm^3^)	125.37 (7.89%)		
Mean cross-validation residual (cm^3^)	− 42.72 (− 2.66%)		
Durbin–Watson statistic	2.18		

	Model-development group	Cross-validation group (range)	Between-session difference (TEM)
Body mass (kg)	78.6 ± 8.2	71.0 (65.7–75.4)	
Height (m)	1.80 ± 0.06	1.82 (1.72–192.8)	
Biceps femoris short head at 75% of thigh length (cm)	2.9 ± 0.6	3.4 (2.3–4.0)	0.00 (0.2%)
Thigh length (m)	0.41 ± 0.04	0.42 (0.39–0.47)	
Knee flexor muscle volume (cm^3^)	1589.5 ± 149.6	1607.2 (1269.3–1826.0)	

Model-development group *n* = 13, cross-validation group *n* = 3

### Ankle plantarflexors

**Fig. 8:**
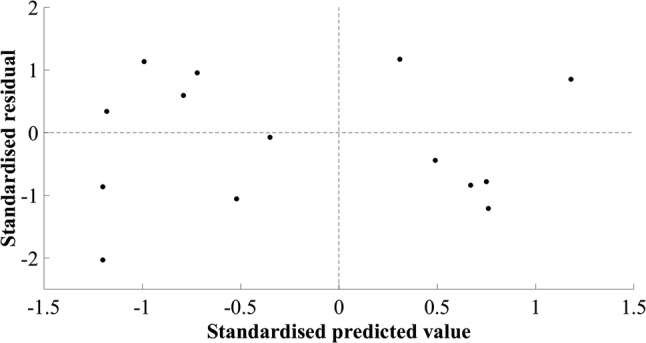
Scatter plot of standardised residual versus standardised predicted values for the final ankle plantarflexor model; 878.606 + (0.553 × lateral gastrocnemius cross-sectional area at 25% of shank length.body mass)

**Fig. 9:**
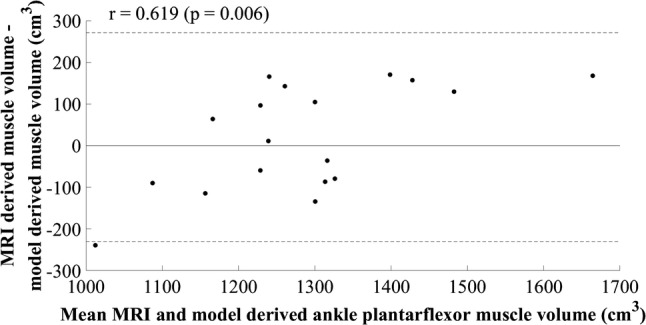
Mean value of ankle plantarflexor muscle volume calculated using MRI and the newly developed model plotted against the difference between methods

One model was identified for the ankle plantarflexors (Table 1). The independent variable was the product of lateral gastrocnemius CSA at 25% of shank length and body mass, in centimetres squared and kilograms, respectively (Table 5). The SEE was 134.91 cm^3^, 10.78%, and the cross-validation residual was 92.44 cm^3^, 6.37%. The Durbin–Watson statistic was larger than the upper limit specified by Durbin and Watson (1951), suggesting that errors were independent. Low standardised residuals were observed throughout the model-development and cross-validation groups and despite the *R*^2^ and adjusted *R*^2^ values being similar (*R*^2^ = 0.387, adjusted *R*^2^ = 0.336), indicating good generalisation of the model, they were low. A positive trend was observed in the plot of standardised residuals versus standardised estimates (Fig. 8), indicating overestimation of small and underestimation of large plantarflexor MV. A multiple regression model resulted in 95% confidence intervals for the constant and lateral gastrocnemius CSA at 25% of shank length crossing zero, indicating poor generalisation. The simple regression model was selected as the final model. The plot of residuals versus the mean value showed a statistically significant positive trend (*r* = 0.619, *p* = 0.006, Fig. 9).

**Table 5:** Ankle plantarflexor muscle group regression equation, model parameters, diagnostic statistics, participant characteristics (mean ± standard deviation), and between-session reliability of ultrasound measurements

Equation ${\text{Ankle plantarflexors muscle volume}}\; = \; 8 7 8. 60 6\; + \;\left( {0. 5 5 3\; \times \;{\text{lateral gastrocnemius cross}}\; - \;{\text{sectional area at 25}}\% {\text{ of shank length}}.{\text{body mass}}} \right)$			
	*β*	Standard error (lower limb–upper limit)	*p* value
Constant	878.606	140.417 (572.662–1184.549)	<0.001
Lateral gastrocnemius cross-sectional area at 25% of shank length.body mass	0.553	0.201 (0.115–0.991)	0.018
*R*^2^	0.387		
Adjusted *R*^2^	0.336		
Standard error of the estimate (cm^3^)	134.91 (10.78%)		
Mean cross-validation residual (cm^3^)	92.44 (6.37%)		
Durbin–Watson statistic	1.44		

	Model-development group	Cross-validation group (range)	Between-session difference (TEM)
Height (m)	1.81 ± 0.07	1.80 (1.72–1.88)	
Body mass (kg)	78.5 ± 7.8	84.1 (67.2–106.5)	
Lateral gastrocnemius cross-sectional area at 25% of shank length (cm^2^)	8.6 ± 1.9	10.2 (9.0–11.9)	0.24 (2.5%)
Ankle plantarflexor muscle volume (cm^3^)	1252.0 ± 165.5	1451.5 (1198.8–1748.2)	

Model-development group *n* = 14, cross-validation group *n* = 4

## Discussion

This study aimed to identify a straightforward tool for quantifying MV of the hip extensor, knee flexor and extensor, and ankle plantarflexor muscle groups, so that MV can potentially be used when monitoring individuals during training or rehabilitation. Generalisable models with normally distributed and independent residuals were developed for each muscle group using reliable and practically applicable independent variables (Table 1). Further work is necessary, however, to improve the models developed for the knee flexors and ankle plantarflexors as systematic error was observed (Figs. 7 and 9). The knee flexor model, as hypothesised, showed reduced accuracy, but the performance of the ankle plantarflexor model led to the rejection of the initial hypothesis that this would be a straightforward muscle group volume to estimate. As expected, the knee extensor model performed well, as did the hip extensor model, and as hypothesised, the latter did not include an ultrasound measurement of muscle size, due to the difficulty in obtaining a representative measurement in a clinically applicable manner. The models developed for the hip and knee extensors have shown good potential for practical application and body mass was an important predictor of volume for the hip and knee extensor and ankle plantarflexor muscle groups, as originally hypothesised.

The final model identified for the hip extensors included the product of body mass and thigh length, and was the only model that did not include a measurement obtained using ultrasound images. This suggests that the ultrasound-acquired MT measurement of gluteal MT was not strongly associated with hip extensor MV. The independent variables included in the final model are justified as the hip extensors play an important role in supporting body mass during locomotion and weight-bearing activities, and over 50% of the muscles in the hip extensor group have insertions on the femur (i.e., the hamstrings, adductor magnus), and the volume of these muscles is, therefore, associated with thigh length. This has implications for applying the model in clinical populations such as above knee amputees or children with cerebral palsy where surgical interventions can lead to a change in the relationship between thigh length and muscle length. In addition, participation in weight-bearing activities, notably independent ambulation, by the recreationally active participants in the current study may have influenced the role of body mass in the hip extensor, knee extensor, and ankle plantarflexor models. Alternative measurements for inclusion in the regression model require identification when considering clinical populations. This can potentially be achieved by considering the acquisition of additional ultrasound measurements. An MT measurement of gluteus maximus has previously been acquired in elderly females (Ikezoe et al. 2011), females with hip osteoarthritis (Fukumoto et al. 2014), and physically active females (Nunes et al. 2018). The larger muscle size in recreationally active males was a limitation to the acquisition of this measurement in the current study as the ultrasound probe could not achieve good quality images of the deep aponeurosis of gluteus maximus. Atrophy is frequently observed in clinical populations, making MT smaller, and a gluteus maximus MT measurement is likely to be obtainable. This is worthy of exploration in future research to work towards improving the performance and application of the hip extensor volume model.

It was not an aim of the current study to report the agreement between ultrasound and MRI acquired CSA measurements. However, the difference in limb position when acquiring images using each of the modalities is a potential limitation to such an analysis. Previous authors have reported differences in ultrasound and MRI CSA despite standardising the image acquisition position (Kruse et al. 2017). In the current study, ultrasound images were acquired in prone, whereas MRI images were acquired in supine where the posterior muscles in the hip extensor, knee flexor, and ankle plantarflexor groups were mildly compressed on the examination table. This may have changed the shape of the muscle in the axial plane, but as muscles are of finite length, one would not expect this to cause a large difference in CSA measurement, in cm^2^. This assumption was proved sound by the inclusion of lateral gastrocnemius CSA at 25% of shank length in the final model for the ankle plantarflexors. The mean absolute difference in lateral gastrocnemius CSA between modalities was 0.60 ± 1.56 cm^2^, similar to previously reported differences in healthy males and females (mean difference: 0.40 ± 1.00 cm^2^, Scott et al. 2012). Ultrasound-acquired CSA has previously been preferred to MT in the estimation of MV of the individual gastrocnemii in children with cerebral palsy (Park et al. 2014), although its widescale application as a measurement tool is limited as panoramic ultrasound is required to obtain a CSA measurement and this is not currently a commonly used clinical tool, despite between-session reliability being established (Tanaka et al. 2017). Therefore, MT may be a more practically applicable measurement, although attempts to include MT in the ankle plantarflexor models in the current study resulted in 95% confidence intervals for the beta coefficients crossing zero, limiting model generalisation. MT has been used to estimate the volume of the plantarflexor muscles in the previous research (Miyatani et al. 2004; Park et al. 2014), although detailed diagnostics are not available and further work is needed to consolidate the plantarflexor MV estimation model. This should include body mass, as in the current study where it was also included in the final model for the hip and knee extensors, reflective of the role of these muscle groups in supporting body weight during locomotion and weight-bearing activities, as opposed to the knee flexors which provide stability.

The practical application of the models developed is dependent on their sensitivity. The final models for the knee extensors and flexors did not display sufficient sensitivity to identify between-leg differences in MV previously reported in male athletes participating in sports with a leg preference, where mean differences of 0.4% and 7.0% have been reported, respectively (Tate et al. 2006). However, the hip extensor model provided sufficient sensitivity to observe previously reported differences in gluteal MV between elite swimmers and controls (mean difference = 14.87%; Semciw et al. 2016), hip extensor MV between sprinters and non-sprinters (mean difference = 21.22%; Handsfield et al. 2017), gluteal MV in controls and late stage hip pathology patients (mean difference = − 12.00 to − 21.00%; Grimaldi et al. 2009a, b), and gluteal MV in amputated and intact limbs in a transfemoral amputee (difference = − 36.5%; Jaegers et al. 1995). The knee extensor model provided sufficient sensitivity to identify differences in knee extensor MV between sprinters and endurance runners (mean difference = 17.77%; Bex et al. 2017), sprinters and non-sprinters (mean difference = 22.83%; Handsfield et al. 2017), controls and type 2 diabetes patients (mean difference = 19.41%; Almurdhi et al. 2016), and hypertrophic changes following 12 weeks of knee extensor training (mean difference = 5.60%; Balshaw et al. 2017). For the knee flexors, differences in MV reported between sprinters and non-sprinters (mean difference = 19.31%; Handsfield et al. 2017), and controls and type II diabetes patients (mean difference = 22.59%; Almurdhi et al. 2016) could be identified with confidence using the model identified in the current study. The ankle plantarflexor model showed sensitivity to atrophy observed in chronic ankle instability patients compared to controls (mean difference = 16.62%; Feger et al. 2016), and differences between sprinters and endurance runners (mean difference = 13.25%; Bex et al. 2017). Overall, the models developed in this study are likely to be able to identify atrophy associated with pathology and disease in key lower limb muscle groups. Given the large differences reported in the existing literature, this is likely to extend to identifying between-limb differences in the presence of pathology, although further improvement of the models, using a larger and more varied sample, is required to identify between-limb differences in healthy, able-bodied populations. The potential to identify changes in muscle size following training supports the clinical application of the models for longitudinal monitoring of patient and athlete progress.

The plots of residuals versus the mean value showed a significant (*p* < 0.05) positive trend for both the knee flexor and ankle plantarflexor models (Figs. 7 and 9), suggesting an underestimation of smaller and overestimation of larger MV, both of which implicate the application of the models for patient management. This may not necessarily be an indication that the model parameters are incorrect; however, they may be suboptimal and further development of the models with a larger, more varied sample could optimise performance. In support of this statement for the knee flexors, the plots of standardised residuals versus standardised estimated values did not show any relationship and all assumptions appeared to have been met (Fig. 6). For the ankle plantarflexors, a positive trend was observed in the residuals plot, suggesting heteroscedasticity (Fig. 8). The use of an alternative independent variable such as MT, and/or the acquisition of MT and CSA measurements at a different shank site (i.e., 30% shank length; Miyatani et al. 2004), is suggested as a worthwhile next step in improving the performance and clinical application of this model. The product of maximal CSA and muscle length has been used previously (Vanmechelen et al. 2018), although a standardised location for acquiring maximal CSA may not be feasible to identify, and the use of shank length as opposed to muscle length due to its straightforward acquisition, must be considered where clinical application is concerned. The relationship between limb length and muscle volume is likely to be implicated in clinical populations such as amputees and children with cerebral palsy and the application of the developed equations in such populations should be investigated, so that the necessary modifications can be made to facilitate the use of the developed models across clinical settings.

## Conclusions

This study has contributed to the development of a straightforward clinical tool for safely and rapidly quantifying the volume of important functional muscle groups of the lower limb by identifying and validating prediction equations using B-mode ultrasound images and anthropometric measurements. Models were developed to estimate hip and knee extensor muscle group volumes using body mass and thigh length, and body mass and an ultrasound measurement of anterior thigh MT, respectively. These models displayed sufficient sensitivity to identify injury related atrophy and training related hypertrophy (hip extensors SEE = 283.01 cm^3^, 8.92%, *R*^2^ = 0.690, knee extensors SEE = 121.10 cm^3^, 5.24%, *R*^2^ = 0.707), supporting their application in a clinical setting. However, improvements in model performance are required to provide sufficient sensitivity for quantifying previously reported muscle volume differences between legs in healthy, uninjured individuals. A model has been identified for the knee flexors, using thigh length and an ultrasound measurement of biceps femoris short-head MT at 75% of thigh length, although further improvement of the model is required to eliminate systematic error before it can be applied with confidence in a clinical setting (SEE = 125.37 cm^3^, 7.89%, *R*^2^ = 0.357). For the ankle plantarflexors, a model was developed using body mass and an ultrasound measurement of lateral gastrocnemius CSA at 25% of shank length (SEE = 134.91 cm^3^, 10.78%, *R*^2^ = 0.387), although further research is required to improve the model, beginning with the identification of appropriate sites for acquiring ultrasound measurements of muscle size that can be replicated with readily available clinical equipment. The models developed for all four key lower limb muscle groups show good potential for widescale clinical application with further research required to expand the size and variety of the data set and optimise the model parameters.

## Data Availability

The data that support the findings of this study are available from the corresponding author [DTR], but restrictions apply to the availability of this data, which was used under participant consent in the current study, and so is not publicly available. Data are available from the corresponding author upon reasonable request, although images will not be able to be provided as they contain sensitive participant information.

## Abbreviations

CSA: Cross-sectional area
MRI: Magnetic resonance imaging
MT: Muscle thickness
MV: Muscle volume
SEE: Standard error of the estimate
TE: Echo time
TEM: Typical error of measurement
TR: Repetition time
